# Microangiopathy in Inflammatory Diseases—Strategies in Surgery of the Lower Extremity

**DOI:** 10.3390/life12020200

**Published:** 2022-01-28

**Authors:** Christoph Biehl, Lotta Biehl, Ingo Helmut Tarner, Ulf Müller-Ladner, Christian Heiss, Martin Heinrich

**Affiliations:** 1 Department of Trauma, Hand and Reconstructive Surgery, University Hospital Giessen, 35392 Giessen, Germany; christian.heiss@chiru.med.uni-giessen.de (C.H.); martin.heinrich@chiru.med.uni-giessen.de (M.H.); 2 Medical Faculty Heidelberg, Heidelberg University, 69117 Heidelberg, Germany; lotta.biehl@gmx.net; 3 Department of Rheumatology and Clinical Immunology, Campus Kerckhoff, Justus-Liebig-University of Giessen, Benekestr., 61231 Bad Nauheim, Germany; i.tarner@kerckhoff-klinik.de (I.H.T.); U.Mueller-Ladner@kerckhoff-klinik.de (U.M.-L.)

**Keywords:** systemic sclerosis, lower extremity, vacuum therapy, surgical treatment

## Abstract

Background: Patients with an inflammatory disease frequently develop chronic angiopathy of the capillaries. Due to this pathology, there is an increased rate of complications in lower extremity surgical procedures. It is not uncommon for microangiopathic wound healing disorders to cause deep infections and fistulas, which lead to prolonged courses and hospitalizations. In addition, adhesions and ossifications of the contractile elements occur regularly. This sometimes results in serious limitations of the mobility of the patients. The study aims to present the results of a combination of vacuum and physical therapy. Patient and methods: A retrospective study of six patients with systemic sclerosis undergoing joint-related procedures of the lower extremity between 2015 and 2020 was performed. In addition to characterization of the patients and therapy, special attention was paid to cutaneous wound healing, affection of the fascia and displacement layers, and sclerosis of the muscle and tendon insertion. Results: The characterized structures (skin, tendon, fascia) show pathological changes at the microangiopathic level, which are associated with delayed healing and less physical capacity. Early suture removal regularly results in secondary scar dehiscence. With a stage-adapted vacuum therapy with sanitation of the deep structures and later on a dermal vacuum system, healing with simultaneous mobilization of the patients could be achieved in our patient cohort. Conclusion: In the case of necessary interventions on the lower extremity, such as trauma surgery, additional decongestive measures in the sense of regular and sustained lymphatic therapy and adapted physiotherapy are indispensable.

## 1. Introduction

Rheumatic autoimmune diseases (RAD) have a prevalence of 1–2% of the population in most European countries [1]. To prevent irreversible damage, the disease must be diagnosed early, and adequate drug therapy must be initiated. To facilitate diagnosis, the American College of Rheumatology (ACR) and the European League Against Rheumatism (EULAR) have developed classification criteria for the systemic sclerosis (SSc), which are regularly updated [2]. In addition to clinical examination and serological detection of autoantibodies, changes in the small vessels can often be detected even in the early stages of connective tissue disease, particularly SSc. Nailfold capillaroscopy, in addition to laboratory tests, facilitates early diagnosis without invasive measures and allows differentiation of an early from an active and a late pattern [3]. It is also well suited for estimating the prognosis or stage of connective tissue diseases. In addition, certain capillaroscopic findings are associated with disease severity and predictive of complications such as pulmonary fibrosis [4]. Thus, specific anti-inflammatory therapy can be initiated at an early stage to achieve a more favourable prognosis. If left untreated, SSc is associated with high morbidity and mortality [5]. Fibrocytes and fibroblast-like synovial cells (FLS) are one of the target cells of SSc [6,7].

As disease progresses, fibrosis of the skin and internal organs occurs with loss of elasticity. Furthermore, there is damage to the vascular endothelium [8]. In other connective tissue diseases such as systemic lupus erythematosus (SLE), activation of the complement system, primarily C5, is a cause of thrombotic microangiopathy [9]. This mainly affects the glomeruli of the kidneys, but also the capillaries in muscles and skin [10]. Therapy focuses primarily on the internal organs (especially the lungs and heart) and the prevention of damage [8].

Involvement of the musculature (myositis) and skin (dermatofibrosis) in the disease process can lead to extensive calcinosis with impairment of limb function. Musculoskeletal manifestations are more common than previously thought [11,12]. Here, a distinction must be made between local and systemic forms, as therapy is directed accordingly [13]. SSc with symptomatic calcinosis of the lower extremity is rare; more obvious are pathologies of the fingers [14]. Calcinosis of the lower extremity can, however, result in significant restriction of mobility and thus have a major impact on the quality of life [15].

If patients present with primary sclerotic plaques on the lower extremity as an initial manifestation without further symptoms, these are often misdiagnosed as a local problem such as an atheroma and excised without histopathological analysis. However, quite frequently wound healing disorders with secondary infection and fistula formation lead to hospitalization.

However, orthopedic and traumatological surgeons, in particular, are challenged to recognize the underlying systemic disease and, if necessary, to procure a diagnosis and systemic therapy by a rheumatologist.

Surgically, the reduction of the calcified tissues is still one of the unsolved problems. To protect the contractile elements and myofascial tissue, often only partial removal of the tissue affected by inflammatory damage is successful. At the same time, there is a risk of iatrogenic secondary tissue damage with increased release of calcium crystals. This can result in a foreign body reaction, persistent inflammation, and non-healing ulcers. Furthermore, postoperative hematomas promote a catabolic microsystem that can lead to secondary sarcopenia. The damaged musculature thus loses part of its function. In addition to muscle fiber damage due to calcification and mechanical removal, satellite cells that play an important role in regeneration are also reduced. Proinflammatory cytokines such as interleukin 6 (IL 6) are locally increased [16].

The risk of developing fistulas also increases with early functional exercise in these cases. On the other hand, prompt initiation of physiotherapy is essential for patients to prevent the adhesion of contractile elements and to avoid jeopardizing patient mobility. This is achieved by treatment with approximately physiological motion sequences and resting postures and requires individualized adaptation of treatment [17].

Joint protection measures relieve the joints, and any orthosis that may be required serves to safeguard static or dynamic instabilities. Taken as a whole, the treatments reduce pain.

Motor-functional training focuses on learning and practicing joint-friendly behaviors and strengthening self-awareness and self-correction [18]. Most injured patients develop pathological movement patterns with compensatory movements and overloading of other joints shortly after starting the exercise. Active joint protection means knowing and taking into account load limits.

An orthosis is not usually required, and if necessary, is only temporary. A dynamic orthosis can be used in the case of pronounced interventions with resulting damage to contractile and stabilizing structures. They are intended to support and help improve the movement of the affected tissues and, if necessary, take over (still) restricted functions.

As an additive therapy, lymphatic drainage is primarily suitable, and additionally edema prophylaxis and scar treatment [17].

This study aims to present a therapeutic strategy for surgical and rehabilitative care in the lower extremity of patients with SSc.

## 2. Patients and Methods

Between 2015 and 2020, more than 2000 surgeries on the lower extremity were performed annually in our hospital. Seventy-eight patients that underwent an operation on the lower extremity had a rheumatic autoimmune disease (RAD). Of these, six patients had concomitant preoperatively confirmed systemic sclerosis with microangiopathy. For the retrospective study, the therapeutic measures taken in these patients were recorded and evaluated. All patients gave written informed consent to participate. No approval was necessary.

Microangiopathy refers to organic localized damage to arterioles and capillaries. Thus, it may be associated with partial or complete loss of function.

The inclusion criteria for this study were confirmed SSc and pathology in the form of tumorous calcification in the lower extremity. Patients were excluded if they did not have evidence of manifest SSc in their medical history or if histopathological examination did not provide evidence of SSc. Another criterion for exclusion was the localized form of scleroderma. The patients were predominantly female (female:male = 5:1), with an average age at the time of surgery of 29 (16 up to 52) years. The average time between the onset of symptoms and surgery was 2 (0.5–4) years. The average duration of SSc disease was 7.8 years.

Our patients reported limits in physical activity and mobility due to the large calcific tumors on the legs. For questions about limits on the lower extremity, the SF-36 is too unspecific, so we used our own questionnaire, according the WOMAC score to assess the patients´ situation.

**Figure 1:**
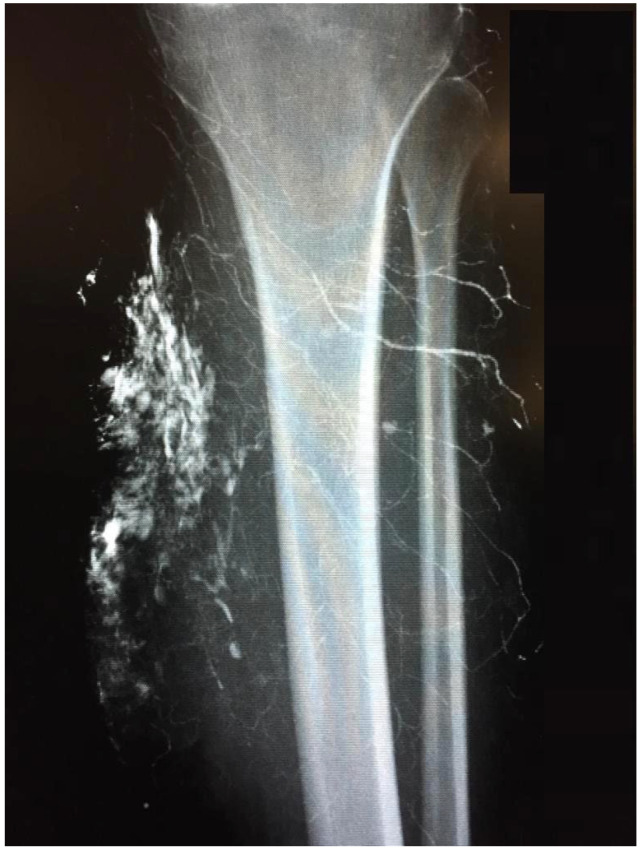
Lymphography of a chronic ulcer of the left tibia. ©UKGM Giessen.

**Figure 2:**
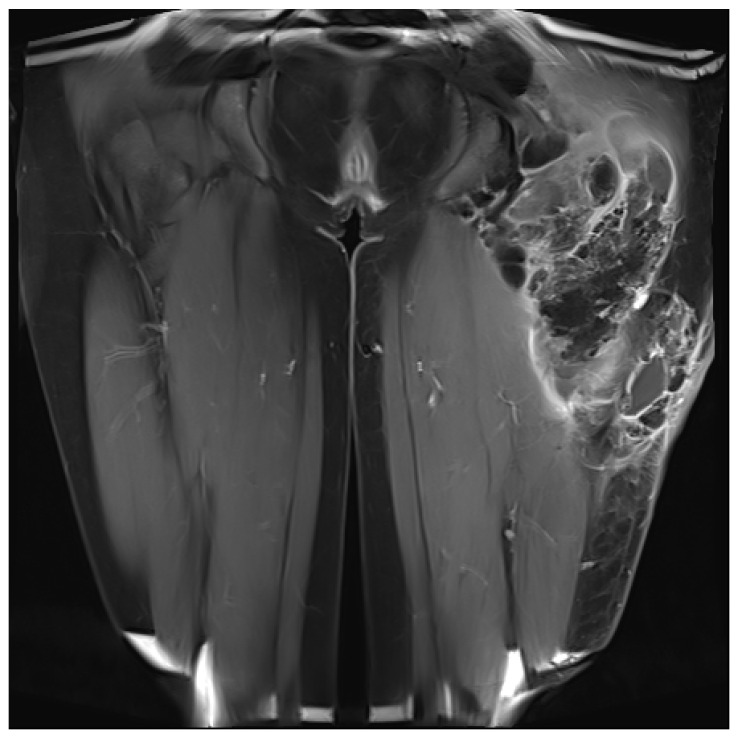
Calcinosis tumor with solid and liquid portions, MRI T2 Sequence. ©UKGM Giessen.

The indication for surgical treatment was a persistent pathology of the musculoskeletal system in the lower extremity. Either because conservative therapy was not successful, or ulcerations were present. Conservative therapy included sufficient medical treatment for RAD and physiotherapeutic therapy and provision of assistive devices where necessary. When surgical treatment was indicated, a preoperative evaluation was performed. Imaging with CT, MRI in conjunction with angiography, and lymphography were used (Figure 1 and Figure 2). Surgeries were performed under steady-state conditions and single-shot antibiotics. The supplies were primarily not joint-related but addressed periarticular tissue.

The primary endpoint was the healing of the calcinotic ulcers. Healing was defined as complete closure of the wound without further secretion, regardless of residual pain.

The secondary endpoint was active mobility of the patients. Follow-up was primarily based on clinical and radiological parameters. It included active and passive mobility of the lower extremity joints in side-by-side comparison and, following the WOMAC score, recording the patients’ subjective limitations [19]. We considered the SF-36 as not appropriate for assessment of the functional situation of the lower extremity, even though it captures impairments in patients’ psychological experience.

Concerning the situation of soft tissue consolidation in connection with the regaining of mobility. The data were weighted and evaluated according to the clinic. However, due to the small group size and the lack of standard distribution, no reliable statistical statement was possible.

We performed a systematic review and research via PubMed, PubMed Central, and Google Scholar following the Preferred Reporting Items for Systematic Reviews and Meta-analyses (PRISMA) protocol [20]. To search all three databases, we combined medical subject headings (MeSH) terms and keywords for identifying articles. The search included the keywords scleroderma and systemic sclerosis (SSc). Our search was narrowed down using the keywords operation, lower extremity. From an initial total of more than 110,000 articles, up to 76 were left, of which eight were included in this manuscript.

## 3. Results

In six patients with systemic sclerosis, 13 surgical interventions were required totally, including two revisions after primary wound healing with new drainage insertions and dermal vacuum therapy.

**Figure 3:**
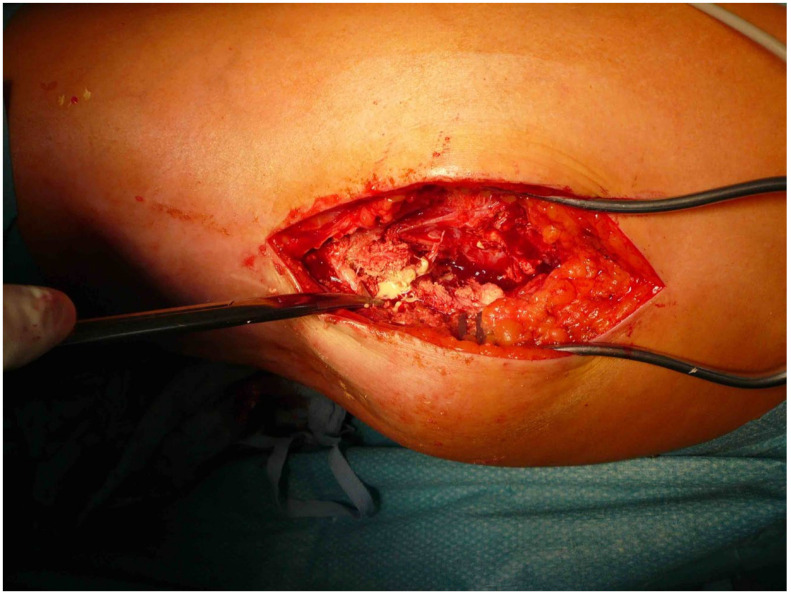
Intraoperative situs of a tumoral calcinosis with solid and liquid portions. ©Dr. I. Tarner, Bad Nauheim.

The patients presented with calcifications in the large muscle groups (gluteal, quadriceps, and gastrocnemius muscles). These spread to tendons and subcutaneous tissue (Figure 2). Four patients had previously undergone trial biopsies, and three of these four subsequently developed a fistula with secondary bacterial colonization of the wound. Surgical therapy was based on the extent of the lesions and consisted of the most radical exstirpation of the affected tissue. In these cases, solid tumor-like calcifications were usually found adjacent to pasty changes (Figure 3).

**Figure 4:**
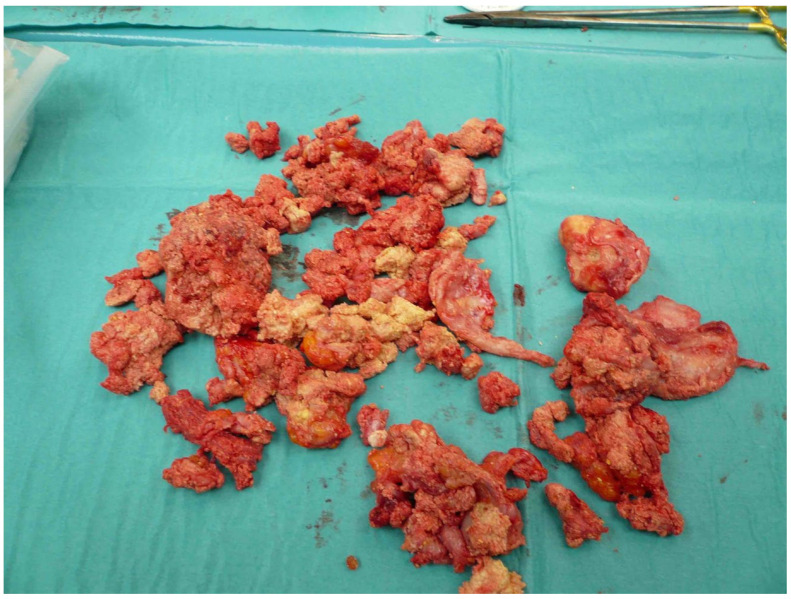
Resectate from the thigh (see Figure 2). ©Dr. I. Tarner, Bad Nauheim.

The removed calcifications weighed up to 700 g (Figure 4). The tissue samples were processed microbiologically and histopathologically. An average of three operations was required to achieve healing of the precarious skin conditions. Two patients developed serous retention postoperatively, requiring repeat drainage insertion and vacuum therapy. Improvement could be achieved by systemic antibiotic application appropriate for resistance, vacuum therapy, and additional prolonged drainage therapy over 3–4 days. This treatment included a staged vacuum therapy with initial sanitation of the lesions, subfascial insertion, and immobilization for 5–7 days. In a later operation, closure of the fascia and Prevena™ therapy for 7–10 days was performed. Of note, the adjacent Prevena™ allowed mobilization of the patient with the help of physiotherapy on walking aids. Initially, the focus was on stabilizing elements and gait training. As healing progressed, these measures were expanded to proprioceptive and nociceptive training. The continuous suction treatment reduces tension in the cutaneous and subcutaneous tissue layers and drains wound edema.

All six patients could be monitored until healing was assured. Two patients are still under regular follow-up. In all patients, healing was achieved. Mobility was ensured at the same time. All patients reported limitations in physical activity and mobility due to the large calcific tumors on the legs, on hospital admission, and during the therapy process. In addition, they reported clear signs of fatigue. In the synopsis of the collected data, this resulted in a decreased quality of life.

Complications: One patient developed a subfascial seroma with renewed fistula postoperatively, which could be healed via puncture/drainage insertion followed by outpatient vacuum therapy over 3 weeks.

## 4. Discussion

Our study shows that surgical treatment of calcific lesions with systemic antibiotics and additional stage-adapted vacuum therapy promotes critical wound healing in SSc patients. It also allows early physical therapy, which benefits mobility. This improves and protects the situation of the internal organs.

Lower extremity calcinosis and myositis can lead to a limitation in life expectancy if there is concomitant evidence of elevated CK [21]. In these cases, Ranque et al. recommend regular heart monitoring, as they found an association of myocardial and skeletal myopathies in such patients [22]. In our patient collective, CK was not elevated preoperatively (<80 U/L). We attribute the postoperative values of up to 174 U/L (normal values 25–140 U/L) to the surgery-related muscle trauma.

However, depending on the extent of the lesions, they sometimes cause significant limitations in mobility and stiffness with concomitant pain [19]. As a result, quality of life is reduced significantly. Del Rosso was able to demonstrate that the results in the Short Form Health 36 (SF-36) in patients with SSc reflect the current psychological state in addition to functional impairments [23]. Furthermore, it provides information about patients’ ability to cope with the health-related quality of life (HRQOL) changes and lifestyle adjustments caused by the disease [24]. For us, it was primarily a question of functional improvement; the restriction of the quality of life was the consequence. According to Angst et al., functional improvements can be better identified with the WOMAC than with the SF-36 [25].

Many authors report impaired or delayed wound healing as the main problem on the lower extremity associated with SSc [26]. In particular, little progress seems to have been made in chronic wounds and fistulas since Hunt in 1936 [27]. Other authors have reported improvement in healing by transplantation of mesenchymal stem cells (MSCs) or fat cells in patients with SSc [28,29]. However, laboratories with experience in cell culture of MSCs or adipocytes could contribute to individualized therapy. To assess which patients might benefit from such therapy, patient safety indicators (PSI) should be collected, as described by Shanmugam et al. for postoperative wound dehiscence in abdominal surgery [30]. The poor healing tendency at the lower extremity depends on vasculitis and microangiopathy of the skin and subcutaneous tissue. The heterogeneity of factors causing SSc makes simple solutions difficult [31,32]. If additional macrovascular damage occurs during the disease, amputation may become inevitable. The duration of SSc disease until amputation was about 13 years in the study by Bertolino et al. and had an additional history of digital ulcerations. Our patients had a shorter history of SSc, averaging 7.8 years. The additional parameters determined by the group were inconstant in our collective, so we do not allow ourselves to draw a conclusion [33]. Shanmugam et al. demonstrated anti-phospholipid antibodies and genetic prothrombotic conditions in a high percentage of patients with lower extremity ulcers [34]. According to Klein and colleagues, patients may also develop a hypercoagulable state with a predisposition to thromboembolic events related to underlying neutropenia and leukopenia [35]. At the same time, certain antirheumatic drugs can cause leukopenia [36]. Therefore, according to current guidelines, perioperative interruption of immunomodulatory drugs is recommended in elective surgery to improve cellular defense [37].

Many patients with known SSc are treated prophylactically with acetylsalicylic acid to improve microcirculation. The challenge for traumatologists is in a short time between trauma and initial care. In addition, there is usually ongoing drug therapy. Patients are admitted after biopsies and resulting fistula that already shows signs of infection. In our cohort, the microbial detection was positive, and dermal mixed flora was found. Reducing the bacteria load in conjunction with reducing calcification was the primary treatment. In addition, systemic antibiosis was given. Because of the difficult skin conditions, as little surgery as possible should be performed. With a staged concept, it is possible to react flexibly to the intraoperative situation and the results of the microbiological examination. Our patients are an example of the fact that the previously propagated concept of staged irrigation very quickly reaches its limits in patients with precarious skin situations. Repeated surgical revisions usually lead to a defective skin situation that can only be solved with complex skin grafts. Rotational flaps or transplantation of full-thickness or split-thickness skin should be avoided because of the difficulty of revascularization. Evaluation of hematologic parameters is also useful to differentiate hemato-oncologic disease and helps prevent recurrence [38].

The problem of limited mobility in SSc is not solved yet. Early mobilization jeopardizes wound healing due to increased mechanical stress. Here, the focus is on two problems. First, in the case of deep infection, and besides systemic antibiotic therapy, a deep subfascial vacuum system is needed [39]. In addition, there is a consecutive significant restriction of mobility of the patient with resulting impairment of internal organs. Secondly, vascular changes compromise the sliding tissues, such as tendons and fasciae. The microstructural changes of skin and subcutaneous tissue show themselves to be very vulnerable in this case. Prolonged retention leads to foreign body-like effects with the additional risk of formation of deep fistulas. Hafner et al. recommend surgical improvement of the vascular situation as a key to wound healing [40]. For microangiopathy predominant in SSc, early intravenous therapy with calcium antagonists improved healing of ulcers [8]. Vacuum therapy improves vascularization and tissue perfusion by stimulating neoangiogenesis. The focus of physical therapy in the first postoperative phase is on decongestive measures, particularly in the form of lymphatic drainage and kinesio-lymph taping. Early functional strength or endurance training is beneficial to prevent a loss of muscle mass and to keep functional deficits as low as possible [41]. The achievable positive effect on muscle mass and strength also has a positive effect on physical and mental performance. Our experience in this regard is in line with the team around Rannou [42]. Physiotherapeutic exercises of muscles and sliding tissue with deep lying drainage systems are limited. The deep vacuum sponges cause significant damage to the muscles by attaching to the muscle fibers and tearing them during forced movements and exercises. Our patients reported increased pain with appropriate physiotherapy and showed poorer psychological resilience in dealing with the condition and the required therapy. Only when the dermal vacuum system is in place is mobilization less risky for the deep muscle and tendon layers possible. In addition to isometric and isotonic exercises, we integrated proprioceptive and nociceptive units at an early stage after an operation. The goal was and is to regain the individual gait pattern. Compensatory movements and relieving postures should be prevented. The combination of intensified physiotherapy and lymphatic drainage was continued in the outpatient setting.

## 5. Limitations

The study suffers from a small number of patients. In addition, the lack of standardization of score collection and laboratory data complicate statistical analysis. The therapy is very individual patient-related, which reduces comparability. Comparison groups of patients with calcinotic ulcers are not available; here, a prospective study may provide valuable insights into the different groups and underlying diseases (RAD, osteoarthritis, diabetics, etc.).

## 6. Conclusions

Ulceration of the lower extremity may occur in patients with manifest SSc. The healing potency depends on micro- and macrovascular changes. Therapeutically, delayed wound healing, fistula, and bacterial infections must be expected. Newer approaches include vacuum therapy through the skin to relieve tissue pressure and the prospect of individualized therapy with bone marrow-derived MSCs in the surgical site. Whether these methods will become a gold standard in lower extremity surgery needs to be confirmed by studies with larger numbers of patients.

## Data Availability

The datasets used and/or analysed during the current study are available from the corresponding author on reasonable request.

## Abbreviations

ACPA	anti-citrullinated protein antibodies
ACR	American College of Rheumatology
CK	creatine kinase
DMARDs	Disease Modifying Anti-Rheumatic Drugs
EULAR	European League Against Rheumatism
FLS	fibroblast-like synovial cells
HRQoL	health-related quality of life
MSC	Mesenchymal Stem Cells
RA	Rheumatoid arthritis
RAD	Rheumatic autoimmune diseases
SLE	systematic lupus erythematosus
SF-36	Short Form Health 36
SSc	systemic sclerosis
WOMAC	Western Ontario and McMaster Universities Osteoarthritis Index
